# Role of Angiopoietic Coronary Endothelial Dysfunction in the Pathogenesis of Ischemic Cardiomyopathy

**DOI:** 10.3390/biomedicines11071950

**Published:** 2023-07-10

**Authors:** Svetlana P. Chumakova, Olga I. Urazova, Vladimir M. Shipulin, Sergey L. Andreev, Olga A. Denisenko, Margarita V. Gladkovskaya, Larisa S. Litvinova, Mikhail A. Bubenchikov

**Affiliations:** 1 Pathophysiology Division, Siberian State Medical University, Tomsk 634050, Russia; urazova72@yandex.ru (O.I.U.); olga.muraveinik@yandex.ru (O.A.D.); gladkovskaya0@gmail.com (M.V.G.); 2 Central Research Laboratory, Siberian State Medical University, Tomsk 634050, Russia; 3 Department of Complex Information Security of Computer Systems, Tomsk State University of Control Systems and Radioelectronics, Tomsk 634050, Russia; 4 Cardiovascular Surgery Unit, Cardiology Research Institute, Tomsk National Medical Research Center, Russian Academy of Sciences, Tomsk 634050, Russia; shipulin@cardio-tomsk.ru (V.M.S.); anselen@rambler.ru (S.L.A.); 5 Immunology and Cell Biotechnology Center, Immanuel Kant Baltic Federal University, Kaliningrad 236041, Russia; larisalitvinova@yandex.ru; 6 Department of Theoretical Mechanics, National Research Tomsk State University, Tomsk 634050, Russia; michael121@mail.ru

**Keywords:** angiogenesis, growth factors, endothelial progenitor cells, coronary heart disease, heart failure

## Abstract

Background: The angiopoietic endothelial dysfunction in ischemic cardiomyopathy (ICMP) remains unexplored. Aim: The identification of the imbalance of endothelial dysfunction mediators and the number of endothelial progenitor (EPC) and desquamated (EDC) cells in patients with coronary heart disease (CHD) with and without ICMP. Methods: A total of 87 patients (47 with ICMP and 40 without ICMP) were observed. The content of EPCs (CD14^+^CD34^+^VEGFR2^+^) in vein blood and EDCs (CD45^−^CD146^+^) in the blood from the coronary sinus and cubital vein was determined by flow cytometry. The contents of HIF-1α and HIF-2α in vein blood as well as that of ADMA and endothelin-1 in sinus plasma and angiopoietin-2, MMP-9 and galectin-3 in both samples were assessed using ELISA, and VEGF, PDGF, SDF-1 and MCP-1 contents using immunofluorescence. Results: ADMA and endothelin-1 levels in the sinus blood were comparable between the patient groups; a deficiency of HIF-1α and excess of HIF-2α were detected in the vein blood of ICMP patients. The EDC content in the vein blood increased in CHD patients regardless of ICMP, and the concentrations of VEGF-A, VEGF-B, PDGF, MCP-1, angiopoietin-2, and MMP-9 were normal. In ICMP patients, vein blood was characterized by an excess of galectin-3 and sinus blood by an excess of EDCs, angiopoietin-2, MMP-9 and galectin-3. Conclusion: ICMP is accompanied by angiopoietic endothelial dysfunction.

## 1. Introduction

Ischemic cardiomyopathy (ICMP) is a serious illness without currently available specific pharmacotherapy. In a proportion of patients, it is characterized by disease progression after the surgical correction of coronary lesions and left ventricular reconstruction [1,2]. This demonstrates the insufficient knowledge of ICMP pathogenesis, in which the role of chronic inflammation, apoptosis of cardiomyocytes, disorders of Ca^2+^ homeostasis and contractile dysfunction of the myocardium, synthesis of various types of collagens and microvascular dysfunction are actively discussed at present [2,3,4]. At the same time, the interest of scientists is focused on the vasomotor form of endothelial dysfunction [5,6]. It is known that asymmetric dimethylarginine (ADMA) uncouples the interaction of NO synthase with arginine in endotheliocytes and reduces NO production, while endothelin-1 is a potent vasoconstrictor that reflects the severity of myocardial ischemia [5,7]. However, the angiogenic form of endothelial dysfunction in ICMP, including angiogenesis disorder and the balance of reparative and destructive processes in the vessels [8], remains without attention.

Since the morphological substrate of coronary heart disease (CHD) and subsequent ICMP is atherosclerosis, both forms of chronic CHD are accompanied by the intimal damage of vessels. Plaque macrophages maintain chronic inflammation and prolong vascular alteration and endothelial desquamation with the help of matrix metalloproteinases (MMPs) [1,9,10], while also contributing to atheroma vascularization, which increases the risk of intraplaque hemorrhage followed by subsequent destabilization [6,11]. On the other hand, angiogenesis induction is necessary for the formation of collateral blood flow and repair of damaged vessels, which has a compensatory protective and adaptive role in CHD and ICMP. Angiogenesis is conducted by endothelial progenitor cells, most of which have a monocytic immunophenotype and reparative potential in relation to the endothelium due to the paracrine secretion of angiogenic factors [12].

In this regard, the study of angiogenesis mediators (such as vascular endothelial growth factor (VEGF) −A and −B, platelet-derived growth factor (PDGF), angiopoietin (Ang)-2, macrophage chemotactic factor (MCP)-1 and stromal cell-derived factor (SDF)-1), along with galectin-3 and MMP-9 production in the heart [12,13] can help to establish the mechanisms of angiogenesis and angiopoietic endothelial dysfunction in CHD with and without ICMP. At the same time, the comparison of the monocytic immunophenotype endothelial progenitor cells (EPCs) and endothelial desquamated cells (EDCs) levels in the blood allows the determination of the adequacy of the angiogenesis mechanisms in relation to the severity of endothelial damage within coronary vessels, and the hypoxia-induced factors HIF-1 and HIF-2 balance reveal the features of mediator response of cells to hypoxia in ICMP compared to CHD without cardiomyopathy.

Our aim is to reveal the features of the imbalance of angiogenic and vasomotor forms of endothelial dysfunction mediators in the coronary blood flow and hypoxia-induced factors in association with the number of endothelial progenitor cells and endothelial desquamated cells in the blood of patients with coronary heart disease, suffering and not suffering from ischemic cardiomyopathy.

## 2. Methods

### 2.1. Clinical Characteristics of CHD Patients

A single-center, cross-sectional, observational, controlled (case–control) clinical study was conducted from December 2019 to December 2022. There were a total of 87 patients with CHD with NYHA class II–IV angina pectoris and predominantly NYHA class II–III circulatory failure (47 patients with ICMP and 40 patients without ICMP), undergoing treatment at the hospital of the Research Institute of Cardiology of the Tomsk National Research Medical Center (Table 1). All patients had a history of acute myocardial infarction. According to Felker G.M. et al. (2002), ICMP criteria were as follows: reduced left ventricular ejection fraction ≤40% with revascularization in anamnesis, stenosis of two or more epicardial vessels ≥75% or stenosis of the left main or proximal part of the left descending coronary artery ≥75% [14]. The control group consisted of 15 practically healthy individuals (13 men and 2 women, with a mean age of 57.63 ± 8.12 years) without cardiovascular disorder. Taking into account the age of the subjects, we included patients with chronic non-cardiovascular diseases in the compensated state in the control group.

Groups of CHD patients were characterized by the equivalent parameters of body mass index, underlying disease duration, age, gender ratio in the study population and the severity of heart failure and angina pectoris. However, they differed significantly in terms of end-systolic index, left ventricular mass and LV ejection fraction, since a decrease in the latter of less than 40% was a criterion for the diagnosis of ICMP and the distribution of patients into groups (Table 1). The comorbidity pattern was similar in the patient groups, except for a higher incidence of type 2 diabetes mellitus in CHD patients without cardiomyopathy versus patients with ICMP (Table 1). All patients with CHD underwent coronary artery bypass grafting, combined with left ventricular reconstruction in cases of ICMP. Before surgery, patients in both study groups received similar conventional drug therapy for CHD: antianginal therapy with the use of long-acting nitrates, β1-blockers and calcium channel blockers, as well as antiplatelet therapy and statin administration. In general, pharmacotherapy in the groups of CHD patients was similar and conducted in comparable doses, with the exception of the more frequent administration of calcium channel blockers and anticoagulants in the group of CHD patients without cardiomyopathy: 62.5% vs. 0% for calcium channel blockers (*p* < 0.001), and 57.5% vs. 23.4% for anticoagulants (*p* = 0.003). Such a therapeutic approach is explained by the low (less than 40%) left ventricular ejection fraction being the contraindication for calcium channel blocker use. The more frequent anticoagulant prescription in CHD without ICMP may be associated with a greater atherogenesis intensity compared to patients with ICMP, and the involvement of lower extremity vessels.

The exclusion criteria included age exceeding 70 years, the presence of autoimmune diseases, allergies in the acute stage, cancer, hypoplastic and megaloblastic anemia, viral hepatitis, syphilis, HIV infection, preoperative courses of treatment with immunosuppressants or erythropoietin administration, acute infection less than 3 weeks before the study and the patient’s refusal to participate in the study.

The study complies with the ethical principles of the 1975 Declaration of Helsinki and was approved by the local ethics committee of Siberian State Medical University (SSMU) (protocol No. 7981 of 16 December 2019). All patients signed an informed consent to participate in the study.

### 2.2. Research Material

Heparinized blood (25 IU/mL) samples from the cubital vein (peripheral blood) and from the coronary sinus (sinus blood) were used as the study material. Peripheral blood was taken in a volume of 5 mL from the cubital vein in healthy individuals after an overnight fast and in all patients on the day of surgery immediately before the induction of anesthesia. Blood from the cubital vein was used for endothelial progenitor cells (EPCs) and endothelial desquamated cell (EDC) immunophenotyping, and its plasma was used for the studied mediators’ concentration assessment. Blood from the coronary sinus in a volume of 5 mL was obtained only from CHD patients intraoperatively, by puncture of the coronary sinus after sternotomy and before initiating the cardiopulmonary bypass. The content of circulating endothelial cells was determined in sinus blood, and the blood plasma from the coronary sinus was used to evaluate the studied mediators’ concentration.

### 2.3. Immunophenotyping of EPCs and EDCs Using Flow Cytometry

The absolute EDC content and the relative EPC content in the blood were determined by flow cytometry in the blood obtained from CHD patients in both study groups and from the cubital vein (peripheral blood) in healthy individuals. In patients with CHD, the EDC content was also assessed in the blood from the coronary sinus. Blood erythrocytes were lysed using FACS Lysing solution (BD Biosciences, Franklin Lakes, NJ, USA); afterwards, the remaining cells were washed 3 times with a Cell-WASH-solution BD buffer (Becton Dickinson, Franklin Lakes, NJ, USA). Mouse Anti-Human CD14-FITC, CD34-PE, VEGFR2 (KDR; CD309)-Alexa Fluor 647, CD45-FITC and CD146-Alexa Fluor 647 monoclonal antibodies were used to determine EPCs with the CD14^+^CD34^+^VEGFR2^+^ immunophenotype and EDCs with the CD45^−^CD146^+^ immunophenotype, according to the manufacturer’s instructions (BD Biosciences, Franklin Lakes, NJ, USA). Thereafter, the cells were kept in the dark with antibodies for 30 min at 4 °C. After that, the cells were washed 2 times in 2 mL of Cell-WASH-solution BD (Becton Dickinson, Franklin Lakes, NJ, USA) and spun down to 250 g for 5 min. Subsequently, 300 µL of Stain Buffer (Becton Dickinson, Franklin Lakes, NJ, USA) were added to the cell pellet and stirred. Fluorescence intensity was determined using Accuri C6 flow cytometer (BD Biosciences, Franklin Lakes, NJ, USA). The scattergrams were plotted and analyzed quantitatively using the BD Cell Quest for Mac OS^®^ X software application version “1.0.264.21 Bulid 20120423.264.21” (BD Biosciences, Franklin Lakes, NJ, USA). The EDC proportion among all the analyzed blood cells was correlated with the total number of leukocytes expressing CD45^+^ (CD45 is a common leukocyte antigen), expressed in ×10^5^/L. The total number of leukocytes in the blood was assessed by flow cytometry using an XS-1000i hematology analyzer (Sysmex Corporation, Kobe, Japan).

### 2.4. Measurement of the Concentration of Mediators in the Blood

Peripheral blood plasma from CHD patients of both study groups and healthy donors, as well as blood plasma from the coronary sinus from CHD patients of both groups, was aliquoted and stored at −80 °C for no more than 12 months. The concentrations of VEGF-A, VEGF-B, PDGF, SDF-1 and MCP1 were determined using a commercial test system for multiplex analysis “Magnetic Luminex Assay Kit for VEGFA, VEGFB, PDGF, SDF1, SCF, FGF1, TGFb1, MCP1” (Cloud-Clone-Corp, Houston, TX, USA) and an automated analyzer Bio-Plex Protein Assay System (Bio-Rad, Hercules, CA, USA). The concentrations of ADMA, endothelin-1, Ang-2, MMP-9, galectin-3, HIF-1α and HIF-2α in the blood plasma were measured by enzyme immunoassay (ELISA), using commercial ELISA kits and following the manufacturer’s instructions: ADMA Xpress ELISA (Immundiagnostik AG, Bensheim, Germany), Human Endotelin-1 ELISA (Bender MedSystems, Wien, Austria), RayBio Human ANGPT2 ELISA Kit (RayBiotech, Peachtree Corners, GA, USA), Human MMP9 ELISA (Thermo Fisher Scientific, Waltham, MA, USA), Human Galectin-3 ELISA (Bender MedSystems, Wien, Austria), Human HIF-1α ELISA Kit (Cloud-Clone-Corp, Houston, TX, USA) and Human HIF-2α ELISA Kit (Cloud-Clone-Corp, Houston, TX, USA). The result was conventionally expressed in pg/mL, while for HIF-2α, it was expressed as the proportion of positive results (HIF-2α was determined) among the subjects of the examined group, since, according to the manufacturer of the test system, HIF-2α is not found in the blood of healthy individuals.

### 2.5. Statistical Analysis of Data

Statistical data processing was conducted with the “Statistica 10.0” software package (JMP Statistical Discovery LLC, Marlow, UK). When describing the results for quantitative characteristics, the median, 25th and 75th percentiles were calculated; for qualitative data, a sample proportion was calculated. Due to the non-normal distribution of sample data for the most indicators, determined according to the Shapiro–Wilk test, non-parametric methods of statistical analysis were used. A comparative analysis of several independent samples was carried out using the Kruskal–Wallis rank test; for the pairwise comparison of the samples, we applied the Mann–Whitney test and the Wilcoxon test using the Benjamini–Hochberg correction for multiple comparisons. To compare the occurrence of a trait frequencies in groups, the chi-squared test was used with Yates’ correction for continuity. To assess the linear relationships between the studied parameters, the Spearman rank correlation coefficient was calculated. The results of statistical analysis were considered significant at *p* < 0.05. The primary data can be accessed via the link https://zenodo.org/record/7760815 (accessed on 22 of March 2023).

## 3. Results

### 3.1. Evaluation of the Balance of Destructive and Reparative Processes in the Coronary Vessels and in the Systemic Circulation in CHD Patients with and without ICMP

In order to study the balance of destructive and reparative processes in the coronary vessels and in the systemic circulation in CHD patients suffering (14 men and 1 woman, aged 61.5 [58.0; 64.5] years) and not suffering (13 men and 3 women, aged 64.0 [57.5; 66.5] years, *p* = 0.218) from ICMP, the EDC and EPC contents in the peripheral blood and the EDC concentration in the blood from the coronary sinus were evaluated.

The EDC content in the peripheral blood of CHD patients, regardless of the ICMP status, exceeded that in healthy donors and did not differ significantly between groups of CHD patients, both in the blood from the cubital vein and from the coronary sinus (Table 2). At the same time, the number of EPCs in peripheral blood in CHD patients without cardiomyopathy was elevated. In patients with ICMP, on the contrary, this parameter varied within physiological values, while the number of EDCs in the sinus blood was 2.5 times greater than that in peripheral blood, which was not observed in CHD patients without cardiomyopathy (Table 2).

### 3.2. The Content of Vasomotor Endothelial Dysfunction Mediators in the Heart and the HIF-Dependent Tissue Response Imbalance to Hypoxia in CHD Patients, Suffering and Not Suffering from ICMP

To detect vasomotor endothelial dysfunction in the coronary vessels in CHD patients, suffering (20 men and 2 woman, aged 60.5 [56.5; 63.0] years) and not suffering (15 men and 4 women, aged 65.0 [58.5; 67.0] years, *p* = 0.436) from ICMP, the ADMA and endothelin-1 concentrations in the blood from the coronary sinus were assessed. To determine the HIF-dependent tissue response balance, the HIF-1α concentration in the peripheral blood and the frequency of HIF-2α detection in the peripheral blood were assessed in both groups of CHD patients.

**Figure 1:**
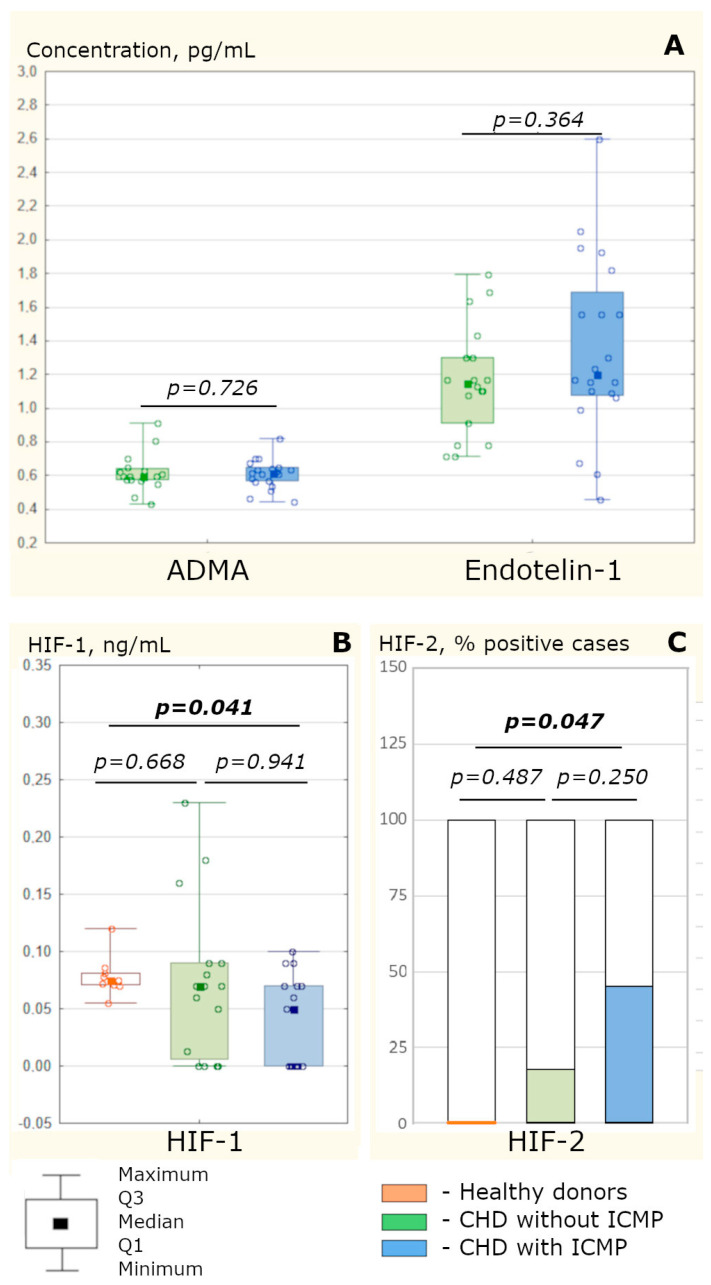
The content of endothelial dysfunction mediators in the sinus blood and hypoxia-inducible factors in the peripheral blood of CHD patients, suffering and not suffering from ICMP. (**A**) The concentration of asymmetric dimethylarginine and endothelin-1 in the sinus blood of CHD patients, suffering from ICMP (*n* = 21) and not suffering from ICMP (*n* = 18), assessed through a Mann–Whitney test. (**B**) The content of hypoxia-inducible factor (HIF)-1α in the peripheral blood of CHD patients, suffering from ICMP (*n* = 17) and not suffering from ICMP (*n* = 16), assessed through a Mann–Whitney test. (**C**) The content of HIF-2α in the peripheral blood of CHD patients, suffering from ICMP (*n* = 20) and not suffering from ICMP (*n* = 17), assessed through a chi-squared test with Yates’ correction. The results are presented as Me [Pe 25; Pe 75], for HIF-2α, as a sample of the proportion of patients showing values above zero. *p* is the level of statistical significance of differences. Notes: ADMA, asymmetric dimethylarginine; HIF, hypoxia-induced factor. The use of color is essential.

The levels of ADMA and endothelin-1 in the sinus blood of CHD patients were comparable between patient groups, demonstrating an almost complete agreement between the values in the patient cohorts (Figure 1A). Meanwhile, the HIF-dependent response of body tissues to hypoxia in CHD patients, suffering and not suffering from ICMP, was different (Figure 1B,C): in cardiomyopathy, HIF-1α deficiency was present, against the background of significantly more frequent HIF-2α detection in the blood (compared to relatively healthy donors); in the absence of cardiomyopathy, only similar trends were determined, without significant differences when compared to healthy donors.

### 3.3. Balance of the Mediators of Angiopoietic Endothelial Dysfunction in the Heart and Systemic Circulation of Patients with Coronary Heart Disease, Suffering and Not Suffering from ICMP

In order to study the balance of the mediators of angiopoietic endothelial dysfunction in the heart and systemic circulation of CHD patients, suffering (39 men and 5 woman, aged 61.5 [57.5; 65.0] years) and not suffering (33 men and 7 women, aged 64.5 [58.5; 68.0] years, *p* = 0.192) from ICMP, the concentrations of SDF-1, MCP-1, Ang-2, MMP-9, galectin-3 and growth factors, namely, VEGF-A, VEGF-B and PDGF, were determined in the blood, sampled from the cubital vein and coronary sinus.

The content of VEGF-A, VEGF-B and PDGF in the peripheral blood of CHD patients was comparable to the values in healthy donors, regardless of the presence of ICMP, and did not differ between groups of patients; however, the analysis of coronary blood flow revealed significant differences (Table 2). Thus, in patients with coronary heart disease without cardiomyopathy, the level of PDGF in the sinus blood was higher, and the concentration of VEGF-B, on the contrary, was lower than in the peripheral blood. At the same time, the content of VEGF-A in the blood from the coronary sinus was higher than that in the blood from the cubital vein in patients with CHD in both groups without differences between the cohorts of patients (Table 2).

An increase in the SDF-1 concentration in the peripheral blood above the level found in healthy individuals was noted only in CHD patients without cardiomyopathy, and the MCP-1 level showed a similar trend (Table 2). In patients with ICMP, the content of both cytokines in the blood from the cubital vein varied within the physiological range and did not differ from the parameters in CHD patients without cardiomyopathy. In the blood from the coronary sinus, the levels of SDF-1 and MCP-1 corresponded to those in the peripheral blood; differences between groups of patients were not detected (Table 2).

The level of Ang-2, MMP-9 and galectin-3 in the peripheral blood of CHD patients without cardiomyopathy did not differ significantly compared to the parameters in healthy donors (Table 2). In ICMP, an excess of galectin-3 in the blood from the cubital vein was detected (with an excess of the level in patients with coronary heart disease without cardiomyopathy) against the background of physiological values of Ang-2 and MMP-9, which did not show differences between groups of patients. Meanwhile, in the blood from the coronary sinus, the concentration of all three mediators in patients with ICMP was higher than in CHD patients without cardiomyopathy. At the same time, galectin-3 level in the sinus blood exceeded that of the peripheral blood in patients of both groups, while Ang-2 concentration was elevated only in patients with ICMP, while MMP-9 content corresponded to its concentration in the peripheral blood, regardless of the presence of ICMP (Table 2).

### 3.4. Interrelationship in the Balance between the Reparative and Destructive Processes in the Endothelium and HIFs-Dependent Mediator Response to Hypoxia with Factors of Angiopoietic and Vasomotor Forms of Endothelial Dysfunction in the Coronary and Systemic Circulation of CHD Patients, Suffering and Not Suffering from ICMP

In order to identify the relationship in the balance between the reparative and destructive processes in the endothelium and the HIF-dependent mediators response to hypoxia with factors of angiopoietic and vasomotor endothelial dysfunction in CHD, a nonparametric correlation analysis was performed between the studied parameters in the blood from cubital vein and coronary sinus in three groups: in a pooled sample of CHD patients (regardless of the presence of ICMP, to identify the universal mechanisms of angiogenesis in CHD) and separately in patients with ICMP and in CHD patients without cardiomyopathy (to identify the features of the angiogenesis mechanisms in CHD with and without ICMP).

**Figure 2:**
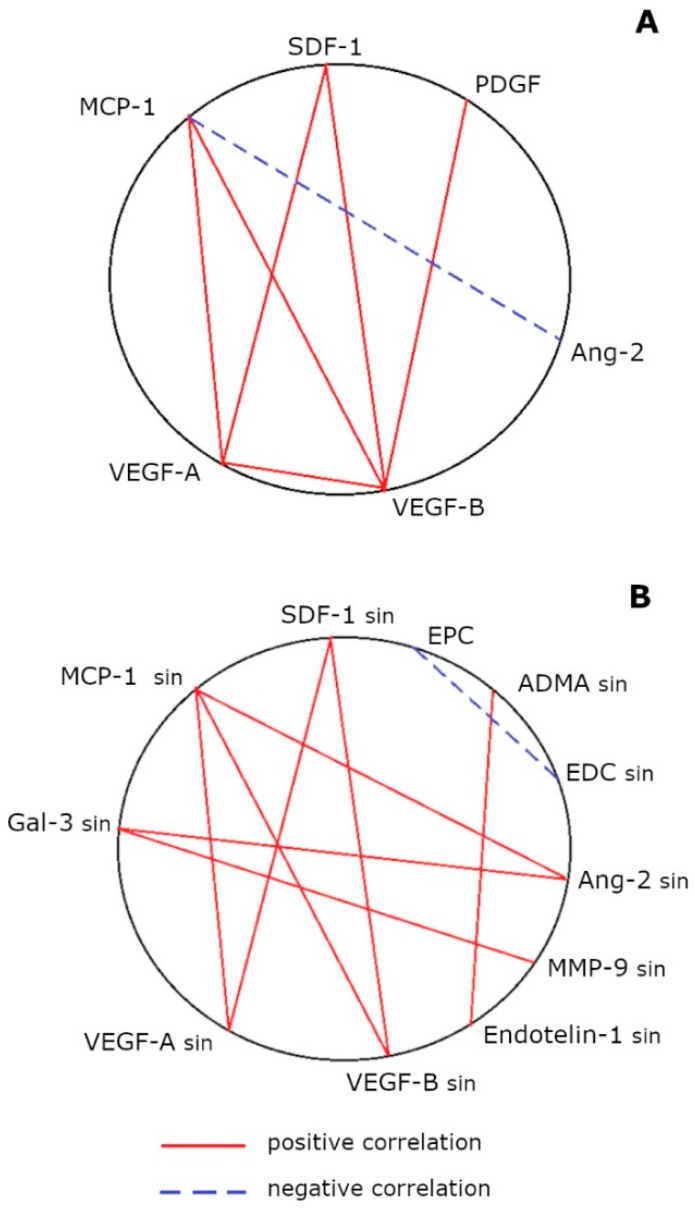
Integral map of the interrelationships between the concentration of angiopoietic and vasomotor endothelial dysfunction mediators and the content of endothelial progenitor cells and endothelial desquamated cells in CHD patients in the pooled sample (regardless of the presence of ICMP) in peripheral (**A**) and sinus (**B**) blood. Notes: ADMA, asymmetric dimethylarginine (*n* = 36); Ang, angiopoietin (*n* = 21 and *n* = 39); EPC, endothelial progenitor cells (*n* = 25); EDC, endothelial desquamated cells (*n* = 21 and *n* = 16); Gal, galectin (*n* = 33 and *n* = 40); MCP, monocytic chemotactic factor (*n* = 59 and *n* = 58); MMP, matrix metalloproteinase (*n* = 32 and *n* = 36); PDGF, platelet growth factor (*n* = 19 and *n* = 17); SDF, stromal cell-derived factor (*n* = 26 and *n* = 21); sin, the parameter was determined in the blood from the coronary sinus; VEGF, vascular endothelial growth factor (VEGF-A: *n* = 53 and *n* = 48; VEGF-B: *n* = 26 and *n* = 20).

In the CHD patients of the pooled sample (regardless of the presence of ICMP) in the peripheral blood, there were positive correlations between the concentration of MCP-1 and VEGF-A (rs = 0.42; *n* = 26; *p* < 0.05), MCP-1 and VEGF-B (rs = 0.60; *n* = 25; *p* < 0.01), SDF-1 and VEGF-A (rs = 0.81; *n* = 26; *p* < 0.001), SDF-1 and VEGF-B (rs = 0.74; *n* = 26; *p* < 0.001), VEGF-A and VEGF-B (rs = 0.75; *n* = 26; *p* < 0.001) and PDGF and VEGF-B (rs = 0, 62; *n* = 18; *p* < 0.001), and a negative correlation between MCP-1 and Ang-2 (rs = −0.67; *n* = 12; *p* < 0.05) (Figure 2A). In the sinus blood of CHD patients of the generalized sample, positive correlations were found between MCP-1 and VEGF-A (rs = 0.51; *n* = 21; *p* < 0.05), MCP-1 and VEGF-B (rs = 0.51; *n* = 20; *p* < 0.05), SDF-1 and VEGF-A (rs = 0.47; *n* = 21; *p* < 0.05), SDF-1 and VEGF-B (rs = 0.60; *n* = 20; *p* < 0.01), MCP-1 and Ang-2 (rs = 0.41; *n* = 31; *p* < 0.05), Gal-3 and Ang-2 (rs = 0.48; *n* = 33; *p* < 0.01), Gal-3 and MMP-9 (rs = 0.42; *n* = 40; *p* < 0.01) and ADMA and endothelin-1 (rs = 0.39; *n* = 33; *p* < 0.05), and a negative correlation between EPCs and EDCs (rs = −0.76; *n* = 11; *p* < 0.01) (Figure 2B).

**Figure 3:**
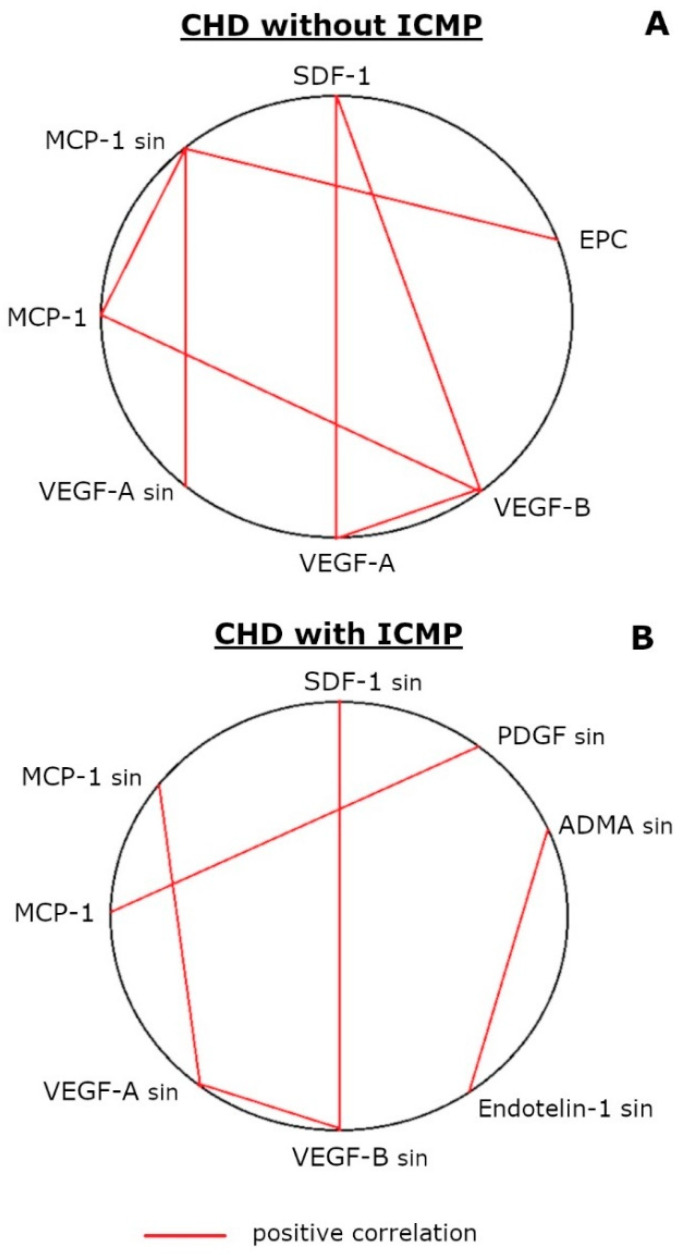
Integral map of the interrelationship between the concentration of angiopoietic and vasomotor endothelial dysfunction mediators and the content of endothelial progenitor and desquamated cells in peripheral and sinus blood in CHD patients, not suffering from ICMP (**A**) and suffering from ICMP (**B**). Notes: ADMA, asymmetric dimethylarginine (*n* = 16 and *n* = 20); Ang, angiopoietin (*n* = 18 and *n* = 23); EPC, endothelial progenitor cells (*n* = 14 and *n* = 11); EDC, endothelial desquamated cells (*n* = 11 and *n* = 11); Gal, galectin (*n* = 19 and *n* = 24); MCP, macrophage chemotactic factor (*n* = 33 and *n* = 36); MMP, matrix metalloproteinase (*n* = 18 and *n* = 24); PDGF, platelet growth factor (*n* = 10 and *n* = 10); SDF, stromal cell-derived factor (*n* = 14 and *n* = 13); sin, the parameter was determined in the blood from the coronary sinus; VEGF, vascular endothelial growth factor (*n* = 26 and *n* = 29).

In the group of CHD patients without cardiomyopathy, positive correlations were found between the level of MCP-1 and EPCs in the sinus blood (rs = 0.72; *n* = 10; *p* < 0.05), the concentration of MCP-1 and VEGF-A in the sinus blood (rs = 0.53; *n* = 15; *p* < 0.05) and MCP-1 in sinus and peripheral blood (rs = 0.72; *n* = 23; *p* < 0.001), as well as between peripheral blood parameters: MCP-1 and VEGF-A (rs = 0.47; *n* = 18; *p* < 0.05), VEGF-A and VEGF-B (rs = 0.90; *n* = 13; *p* < 0.001), SDF-1 and VEGF-A (rs = 0.76; *n* = 13; *p* < 0.01) and SDF-1 and VEGF-B (rs = 0.65; *n* = 13; *p* < 0.05) (Figure 3A).

In the group of patients with ICMP, positive correlations were found between the level of MCP-1 in the peripheral blood and PDGF in the sinus blood (rs = 0.82; *n* = 10; *p* < 0.01), as well as between sinus blood parameters: MCP-1 and VEGF-A (rs = 0.49; *n* = 20; *p* < 0.05), VEGF-A and VEGF-B (rs = 0.81; *n* = 10; *p* < 0.01), SDF-1 and VEGF-B (rs = 0.68; *n* = 10; *p* < 0.05) and ADMA and endothelin-1 (rs = 0.63; *n* = 18; *p* < 0.01) (Figure 3B).

## 4. Discussion

According to the obtained data, in patients with CHD complicated and uncomplicated by ICMP, a significant difference was found in the nature of the angiopoietic dysfunction mediator imbalance in the blood from the coronary sinus against the background of a comparable level of vasomotor from mediators in the coronary blood flow (Table 2 and Figure 1).

### 4.1. The Development of ICMP Is Not Accompanied by a More Pronounced Vasomotor Endothelial Dysfunction of the Coronary Vessels Compared to CHD without Cardiomyopathy

Being a marker of endothelial dysfunction, ADMA is synthesized under hypoxic conditions by inducible nitric oxide synthase (iNOS) and changes NOS activity, contributing to the reactive oxygen species (ROS) production and NO deficiency [5,15,16]. Another vasomotor endothelial dysfunction mediator, endothelin-1, synthesized not only by endotheliocytes, but also by smooth muscle cells, fibroblasts and macrophages, also mediates vasospasm and is positively correlated with the pro-inflammatory cytokine production [5,16]. Meanwhile, the concentration of ADMA and endothelin-1 in CHD patients with and without ICMP did not differ in the blood from the coronary sinus (Figure 1A). This circumstance cannot be associated with the peculiarities of the therapy of the two groups of patients. Despite the fact that beta-blockers, vasodilators, statins and angiotensin-converting enzyme inhibitors can reduce the plasma level of endothelin-1 [5], the groups of CHD patients were comparable in terms of the rates of prescriptions of these drugs (see Section 2.1). Therefore, vasomotor endothelial dysfunction involving ADMA and endothelin-1 plays an equal role in the formation of both CHD with and without ICMP and is not a key factor in the ICMP pathogenesis. At the same time, the mechanisms of these mediators’ formation in the heart are interconnected, which is confirmed by a positive association between the level of ADMA and endothelin-1 in the coronary blood flow (see Section 3.4), which was determined in a pooled sample of CHD patients (Figure 2C) and in patients with ICMP (Figure 3B). This correlation is due to the fact that the expression of endothelin-1 is inhibited by NO, prostacyclin and atrial natriuretic peptide [5,7].

### 4.2. The Coronary Vessels’ Endothelium Desquamation in ICMP Is Increased, Which Is Not Accompanied by the EPC Mobilization from the Bone Marrow Due to the Absence of the Excess of SDF-1 and MCP-1 Chemoattractants in the Blood

Despite the absence of differences in the vasomotor endothelial dysfunction mechanisms in the coronary vessels in CHD patients with and without ICMP, only ICMP patients demonstrated an increase in EDC content in the blood from the coronary sinus compared to the corresponding level in peripheral blood. At the same time, the EPC level in the blood from the cubital vein remained normal. In CHD patients without cardiomyopathy, on the contrary, EDC values in blood samples were comparable, with high levels of EPCs in the systemic circulation (Table 2). This indicates an increased recruitment of EPCs with reparative potential from the bone marrow into the blood in CHD patients without cardiomyopathy, which is a compensatory reaction in atherogenesis and, obviously, provides reparative angiogenesis adequate to the endothelium destruction in the heart. In ICMP, this compensatory reaction, apparently, is not realized: the physiological level of EPCs in the blood is insufficient for the coronary vessels’ repair under atherosclerosis conditions; therefore, angiogenesis is not effective and endothelial destruction predominates, which proves the presence of angiopoietic endothelial dysfunction in ICMP. This is consistent with the negative correlation between the EPC content in the peripheral blood and EDC content in the sinus blood of the CHD patients of the pooled sample (see Section 3.4, Figure 2B). It is important to note that the EDC assessment in the blood from the cubital vein did not reveal an increased coronary endothelium destruction in ICMP (Table 2).

Insufficient coronary vessels’ repair in ICMP may be due to an angiogenic mediator imbalance that ensures EPC mobilization from the bone marrow, their homing and proliferation/differentiation/secretory activity in the coronary vessels. Among the studied angiogenic mediators in the peripheral blood of CHD patients without cardiomyopathy, who demonstrate an excess of EPCs, only SDF-1 was elevated, while in patients with ICMP, both parameters were within the reference range. SDF-1 accumulation in the plasma stimulates CXCR4^+^ cell mobilization from the bone marrow, including hematopoietic stem cells and EPCs, which express CXCR4 as a receptor for SDF-1. The SDF-1 and CXCR4 interaction also stimulates the recruitment and retention of stem cells in ischemic areas [13,17,18,19,20]. Another chemotactic factor, MCP-1, mediates monocytic EPC attachment to the damaged endothelium, which is accompanied by their differentiation into cells expressing endothelial markers (Tie2, CD31 and VE-cadherin) and the inhibition of vascular intimal hyperplasia [21]. This explains the presence of a positive relationship between the MCP-1 level in the sinus blood and the EPC content in the peripheral blood of CHD patients of the pooled sample and of CHD patients without cardiomyopathy (see Section 3.4, Figure 2B and Figure 3B). However, this relationship was absent in patients with ICMP, apparently reflecting the insufficient MCP-1 participation in EPC homing to the coronary vessels in ICMP. Despite the fact that, in the present study, no significant differences were found in the MCP-1 concentration in the blood between samples from the cubital vein and the coronary sinus, as well as between groups of patients with coronary heart disease (Table 2), we previously described the excessive MCP-1 concentration in the sinus blood relative to the peripheral blood of CHD patients without cardiomyopathy, which was not determined in patients with ICMP (MCP-1 level was determined by ELISA) [22]. The present study registered only a similar trend (Table 2), but revealed many correlations of MCP-1 with the concentration of other studied mediators, indicating its important role in angiogenesis.

It is important to note that, in CHD patients without cardiomyopathy, patients with ICMP were less likely to have diabetes mellitus (Table 1), which contributes to endothelial damage; however, the number of EDCs in the peripheral blood was not lower, and in the sinus blood, on the contrary, even greater than in CHD without cardiomyopathy. Therefore, increased intimal damage of the coronary vessels in ICMP occurs not due to glycation of endotheliocyte proteins and their osmotic stress, but with the involvement of other mechanisms. The rare occurrence of diabetes mellitus in ICMP can be explained by an increased level of galectin-3 in the blood (Table 2), which can interact with the insulin receptor and protein glycation products, promoting their utilization, which prevents the development of insulin resistance [22].

### 4.3. HIF-1 and HIF-2 Imbalance in ICMP Does Not Affect VEGF-A Production but Reduces PDGF Production and VEGF-B Consumption in the Myocardium, Disrupting Vessels’ Early Stabilization

HIF-1 is the central regulator of angiogenesis, since it enhances the gene transcription for several proangiogenic proteins (SDF-1, VEGF, PDGFB, Ang-1 and Ang-2) and their receptors [23], thereby preventing ischemic myocardial damage [24]. Despite the presence of heart failure of comparable severity in patients of both groups (Table 1), in CHD patients without cardiomyopathy, the HIF-1α and HIF-2α content did not differ from the parameters of healthy donors, and in patients with ICMP, there was a HIF-1α deficiency with an elevated HIF-2α level (Figure 1B,C). This is probably due to the realization of various types of hypoxias: in CHD without cardiomyopathy, cyclic hypoxia develops (during exercise), while in ICMP, hypoxia is chronic (even at rest). During hypoxia, HIF-1 is activated within the first four hours, after which it decreases, and the maximum HIF-2α level is reached after 24–48 h. In chronic hypoxia, HIF-1α expression decreases under the influence of heat shock protein 70 (Hsp70), which causes the ubiquitination of HIF-1α but not of HIF-2α; pro-inflammatory reactions are also inhibited, since NF-kB competes with HIF-α for the common p300 coactivator [25]. HIF-1α enhances the proliferation and differentiation of cells involved in angiogenesis, and HIF-2α controls vascular stabilization by stimulating the expression of fibronectin (a component of the basement membrane of blood vessels) [25]. HIF-1α deficiency in the blood of patients with ICMP, apparently, determines the absence of compensatory SDF-1 hyperproduction during hypoxia and affects other angiogenic factors’ secretion as well.

The VEGF-A content in the blood from the coronary sinus exceeded that in the peripheral blood of CHD patients of both groups, likely reflecting the induction of angiogenesis under ischemic conditions (Table 2). VEGF-A binds to VEGFR1 and VEGFR2, stimulating EPC proliferation and differentiation into endothelial cells and the formation of tubular structures and increasing vascular wall permeability, and inhibits cardiomyocyte apoptosis [13,26,27,28]. Given that hypoxia increases VEGFR1 expression [27], which is a decoy receptor for VEGF-A, may inhibit angiogenesis [28] and activate MMP-9 secretion from vascular myocytes [29], patients with ICMP, due to chronic hypoxia, may present with an attenuation of the interaction between VEGF-A and VEGFR2. At the same time, in contrast to the proangiogenic VEGF-Axxxa family, there is also VEGF-Axxxb family that inhibits angiogenesis [29]. The synthesis of the latter increases under the influence of TGF-β [29], which is actively secreted in the myocardium of patients with ICMP [22]. In addition, VEGF-A has proatherogenic properties (accumulates triacylglycerols and inhibits lipoprotein lipase), in contrast to VEGF-B, which has hypolipidemic effects (reduces cholesterol absorptio and cholesterol load on the plasma membrane). VEGF-B binds to VEGFR1 and has a weak angiogenic activity, but promotes glucose oxidation, compensatory myocardial hypertrophy and the synthesis of macromolecules, antioxidants and natriuretic factors [28]. Given that VEGF-B is synthesized in the myocardium, myocytes and endothelium of the coronary arteries, pancreas, lungs, kidneys, gallbladder and adipose tissue [27,28], a decrease in VEGF-B concentration in the coronary circulation of CHD patients without cardiomyopathy may be associated with its increased consumption in the myocardium (Table 2). At the same time, an increase in PDGF concentration in the sinus blood relative to the peripheral blood of CHD patients without cardiomyopathy (Table 2) indicates the stabilization of the newly formed vessels in the heart with the participation of VEGF-A, which probably does not occur in patients with ICMP. It is known that PDGF not only promotes differentiation and EPC mobilization from the bone marrow and their migration [30], but also vascular maturation, since, unlike VEGF, it attracts pericytes [31], vascular smooth muscle cells and stimulates the endothelial–mesenchymal transition [32].

### 4.4. In ICMP, a High Content of Ang-2 and MMP-9 without an Adequate Increase in VEGF-A in the Myocardium Has a Destructive Effect on the Vessels Associated with Galectin-3 Hyperproduction in the Heart

Ang-2 is a negative regulator of angiogenesis, since it blocks the binding of pro-angiogenic Ang-1 to their common Tie-2 receptor, destabilizes early vessels and increases their permeability [33]. Their different response to hypoxia has been shown: Ang-2 overproduction is more often observed during ischemia and aggravates it [34], while Ang-1 is observed mainly in malignant neoplasms [35]. However, in the presence of excessive VEGF-A, it can act as a Tie-2 agonist and activate angiogenesis, and in its absence, it is associated with vascular regression [29]. Therefore, an increase in the Ang-2 concentration in the sinus blood of patients with ICMP relative to CHD patients without cardiomyopathy with a comparable VEGF-A level in the coronary circulation (Table 2) can be considered as a sign of angiogenesis impairment in ICMP. Ang-2 and MMP-9 are considered markers of cardiovascular disease, atherosclerosis and endothelial dysfunction [7]. MMP-9 destroys the components of the extracellular matrix, including fibronectin [22,36], which is part of the basolateral vascular membrane [37]. This can promote both angiogenesis [35] and vascular damage [7]. Considering that, in patients with ICMP, the content of MMP-9 and EDCs in the sinus blood was higher than that in the peripheral blood and, in CHD patients without cardiomyopathy, it was found to be equal (Table 2), the MMP-9 hypersecretion in the myocardium probably indicates its angiodestructive effect. It is noteworthy that, in the pooled sample of CHD patients, there was a positive association between the level of Ang-2 and MMP-9 and the galectin-3 level (see Section 3.4, Figure 2B), which is secreted in the heart by macrophages and ischemic cardiomyocytes, and is a marker of myocardial damage, fibrosis and heart failure [22,38]. On the one hand, it promotes the formation of reactive oxygen species and cell apoptosis, while on the other, it initiates the proliferation and migration of endothelial cells, participating in angiogenesis [22,38]. It has been shown that galectin-3 mediates aldosterone-induced perivascular fibrosis, since Gal-3 overexpression specifically enhanced the synthesis of type I collagen in rat vascular smooth muscle cells [39]. This leads to the assumption that the role of galectin-3 in angiogenesis in CHD is more destructive than protective, since ICMP, as its more severe form, was accompanied by an increased content of galectin-3, Ang-2, MMP-9 and EDCs in the coronary circulation (Table 2).

### 4.5. The ICMP Pathogenesis Is Associated with Angiopoietic Endothelial Dysfunction, which Involves an Uncoordinated Mediator Response to Ischemia and Atherogenesis

The integration of correlations (see Section 3.4, Figure 3A,B) allows us to conclude that CHD patients without cardiomyopathy have a coordinated angiogenic response at the organismic level with the involvement of angiogenesis activators VEGF-A, VEGF-B and SDF-1 in the peripheral blood. In this response, myocardial MCP-1 plays a central role: it determines its own level and the increase in the EPC number in the systemic circulation (see Section 3.4, Figure 3A). This is consistent with the data on the ability of MCP-1 to stimulate VEGF-A secretion by removing miR-374b-5p for the latter [40] and EPC homing in tissues [21]. In ICMP, the angiogenic response is uncoordinated and is realized only at the organ level with the participation of VEGF-A, VEGF-B and SDF-1 only from the myocardium, without affecting the EPC number in the blood (see Section 3.4, Figure 3B). Therefore, in ICMP, only humoral factors are involved in angiogenesis, but not cellular ones.

Thus, the development of CHD without cardiomyopathy is accompanied by an increased mobilization of EPCs from the bone marrow in response to atherogenesis under the action of excess SDF-1 in the blood, which are actively recruited to the heart by VEGF-A and PDGF. Mature vessels form in the myocardium due to PDGF secretion in response to cardiomyocyte injury, which is mediated by galectin-3 and causes moderate endothelial desquamation. The ICMP formation is associated with the absence of increased EPC mobilization due to an HIF imbalance in the blood. EPCs are recruited into the myocardium by VEGF-A alone, where immature vessels form without PDGF involvement. Angiogenesis is inadequate to the degree of vascular damage and therefore exacerbates cardiomyocytes ischemia. These events mediate the involvement of Ang-2 and MMP-9 in vascular injury during ICMP, which is associated with an excessive galectin-3 production in the myocardium and forms a vicious circle of myocardial ischemia.

## 5. Conclusions

The leading factor in the ICMP pathogenesis is angiopoietic endothelial dysfunction of the coronary vessels due to the lack of compensatory EPC mobilization enhancement from the bone marrow under conditions of a mild and uncoordinated proangiogenic tissue response. Vasomotor endothelial dysfunction involving ADMA and endothelin-1 in the ICMP development has no distinctive features relative to CHD without cardiomyopathy. The established disorders of the angiogenesis mechanisms in ICMP will allow the determination of the targets for the angiogenic therapy of this aggravating disease.

## 6. Research Limitations

The results of the study may be limited by the clinical status of patients because the data obtained are valid for CHD patients with hemodynamically significant multivessel lesions of the main coronary arteries. Therefore, in patients with ICMP at the initial stage (without manifestations), established patterns may not yet be detected, and thus further research is needed. Since the study population included mainly Caucasian patients from the Siberian Federal District, for other populations of Russia and the global community, some of the identified patterns may differ from the those established in this study.

## Figures and Tables

**Table 1:** Characteristics of CHD patients (suffering and not suffering from ICMP).

Clinical Parameters	CHD Patients without ICMP	CHD Patients with ICMP	*p*
Number of patients:	40	47	-
male	33 (82.50%)	42(89.36%)	0.540
female	7 (17.50%)	5(10.64%)	0.540
Age, years	64.5 [58.5; 68.0]	61.0 [57.0; 64.5]	0.123
CHD duration, years	5.00 [2.50; 9.00]	3.50 [1.00; 6.50]	0.264
Body mass index, kg/m^2^	29.48 [26.33; 32.51]	28.07 [26.69; 31.18]	0.650
Functional class of angina			
II	7 (17.50%)	11 (23.40%)	0.680
of effort			
III	29 (72.50%)	31 (65.96%)	0.671
IV	4 (10.00%)	5 (10.64%)	0.798
Heart failure I NYHA	4 (10.00%)	3 (6.38%)	0.824
Heart failure II NYHA	16 (40.00%)	28 (59.57%)	0.109
Heart failure III NYHA	20 (50.00%)	16 (34.04%)	0.198
LV ejection fraction, %	59.25 [50.00; 67.50]	31.50 [23.25; 36.50]	**<0.001**
Eventual systolic index of LV, mL/m^2^	52.90 [50.20; 56.90]	75.30 [64.30; 82.30]	**0.004**
LV myocardium mass, g	184.0 [140.5; 214.5]	233.0 [221.7; 266.2]	**<0.001**
Statin therapy	34 (85.00%)	39 (82.98%)	0.971
Hypertensive disease stage III	33 (82.50%)	32 (68.09%)	0.196
Type 2 diabetes mellitus	13 (32.50%)	4 (8.51%)	**0.011**
Gastric and/or duodenal ulcer	9 (22.50%)	5 (10.64%)	0.227
Diseases of liver and biliary tract	5 (12.50%)	3 (6.38%)	0.541
Chronic kidney disease	9 (22.50%)	15 (31.91%)	0.460
Pulmonary diseases	6 (15.00%)	8 (17.02%)	0.971

Notes: Results are presented as Me [Pe25; Pe75] or *n* (%). The accepted level of statistical significance was *p* < 0.05 (bolded values). LV, left ventricular.

**Table 2:** The level of EDCs, EPCs, and mediators of angiopoietic endothelial dysfunction in the blood from the cubital vein and coronary sinus in CHD patients, with and without cardiomyopathy, Me [Pe 25; Pe 75].

Blood Parameters	Healthy Donors	CHD Patients without ICMP	CHD Patients with ICMP
Blood from the Cubital Vein (Peripheral Blood)			
EDC, ×10^5^/L	5.12 [3.73; 5.84]	7.25 [6.80; 7.47] **Pc = 0.038**	7.26 [5.43; 17.94] **Pc = 0.037** P_2_ = 0.597
EPC, %	4.10 [2.70; 5.00]	6.63 [4.70; 13.00] **Pc = 0.042**	4.93 [2.20; 7.30] Pc = 0.369 P_2_ = 0.678
VEGF-A, pg/mL	3.80 [1.00; 6.50]	4.50 [3.00; 8.00] Pc = 0.314	6.00 [3.00; 9.50] Pc = 0.216 P_2_ = 0.502
VEGF-B, pg/mL	1.32 [1.00; 3.10]	1.60 [1.27; 2.20] Pc = 0.772	1.30 [1.00; 1.45] Pc = 1.000 P_2_ = 0.570
PDGF, pg/mL	2.68 [1.65; 7.10]	3.10 [2.10; 7.05] Pc = 1.000	4.85 [1.20; 9.10] Pc = 1.000 P_2_ = 0.870
SDF-1, pg/mL	30.00 [5.00; 45.00]	60.00 [50.00; 80.00] **Pc = 0.042**	49.00 [37.00; 56.00] Pc = 0.174 P_2_ = 0.115
MCP-1, pg/mL	190.0 [140.0; 240.0]	210.0 [144.4; 268.0] Pc = 0.612	202.5 [164.0; 324.0] Pc = 0.864 P_2_ = 0.527
Galectin-3, ng/mL	6.50 [5.60; 7.64]	6.10 [4.30; 7.48] Pc = 0.928	8.20 [7.20; 10.00] **Pc = 0.025** **P_2_ = 0.017**
Angiopoetin-2, pg/mL	388.0 [317.0; 460.0]	445.0 [137.5; 552.5] Pc = 1.000	430.0 [380.0; 580.0] Pc = 1.000 P_2_ = 0.971
MMP-9, pg/mL	13.20 [9.60; 19.00]	11.95 [7.00; 13.40] Pc = 0.460	13.65 [6.50; 19.60] Pc = 0.848 P_2_ = 0.588
Blood from the coronary sinus (sinus blood)			
EDC, ×10^5^/L	–	10.17 [6.80; 18.83] P_1_ = 0.128	17.98 [10.27; 22.97] **P_1_ = 0.036** P_2_ = 0.156
VEGF-A, pg/mL	–	7.80 [3.25; 9.75] **P_1_ = 0.041**	6.89 [3.25; 15.60] **P_1_ = 0.007** P_2_ = 0.918
VEGF-B, pg/mL	–	1.00 [0.85; 1.36] **P_1_ = 0.011**	1.02 [0.89; 1.08] P_1_ = 0.285 P_2_ = 0.762
PDGF, pg/mL	–	7.60 [3.70; 9.94] **P_1_ = 0.036**	7.86 [2.92; 8.77] P_1_ = 0.674 P_2_ = 0.736
SDF-1, pg/mL	–	40.30 [26.00; 62.00] P_1_ = 0.086	46.80 [32.50; 64.00] P_1_ = 0.286 P_2_ = 0.623
MCP-1, pg/mL	–	227.5 [135.2; 331.5] P_1_ = 0.209	242.5 [176.2; 321.8] P_1_ = 0.585 P_2_ = 0.638
Galectin-3, ng/mL	–	13.13 [10.14; 15.86] **P_1_ < 0.001**	20.15 [14.17; 60.06] **P_1_ < 0.001** **P_2_ = 0.012**
Angiopoetin-2, pg/mL	–	767.0 [494.0; 988.0] P_1_ = 0.128	1111.5 [845.0; 1235.0] **P_1_ < 0.001** **P_2_ = 0.002**
MMP-9, pg/mL	–	5.92 [5.07; 17.42] P_1_ = 0.972	16.64 [6.63; 29.12] P_1_ = 0.649 **P_2_ = 0.038**

Notes: The level of statistical significance of differences in comparison: Pc, with the control (healthy donors); P_1_, with the content of factors in the peripheral blood in patients of the corresponding study group; P_2_, with patients with coronary heart disease. The accepted level of statistical significance was *p* < 0.05 (bolded values).

## Data Availability

The data presented in this study are openly available in https://zenodo.org/record/7760815 (accessed on 22 of March 2023).
